# 

**Published:** 1892-03

**Authors:** 


### LITERARY.

CONSUMPTION.—HOW TO PREVENT IT, AND HOW TO LIVE WITH IT, etc. By Professor W. S. Davis, Jr. A. M., M. D. 4to, 143 pp. F. A. Davis, Publisher, Philadelphia. Price, 75 cents, net.

This compact little volume had its origin in a series of hygienic rules prepared by the author, for his patients, especially those living at a distance.

The chief elements of good health are pure dry air, moderate exercise, nutritious food, and sufficient clothing.

The author affirms that consumption is not contagious, as it was almost uniformly supposed to be 100 years ago. It is communicated by bacilli, ejected in the sputa of those affected with the disease, to which the bacilli adhere and exist even after it becomes dry and powdered and is blown hither and yon in the wind, hence great care should be observed in its disposition.

The work is carefully written and throws much light upon the prevention and treatment of this scourge of the human race, which infects all lands and spares neither youth nor old age.

We commend it more especially to the young and inexperienced practitioner of the noble art of healing.

NOTES ON TREATMENT OF CATARRHAL INFLAMMATIONS, etc. By Beverly Robinson, M.D., New York.

This is a very clear and comprehensive treatise on catarrhal affections and their nature, and is highly commended by the medical faculty.

TRANSACTIONS OF THE DETROIT MEDICAL AND LIBRARY ASSOCIATION, 1891-2.

The medical essays, composing in great measure the published proceedings of this body, will compare favorably with those of any kindred association within our knowledge.

THE CHINESE.—THEIR PRESENT AND FUTURE: MEDICAL, POLITICAL AND SOCIAL. By Robert Coltman, Jr., M.D. 4to, 212 pp. F. A. Davis, Publisher, Philadelphia.

The author of this admirable volume entered upon the only course which gives value to works of this discription. He attached himself to the missionary establishment of North China and travelled extensively over that interesting region in the capacity of surgeon and physician, studying with great care the manners, customs, and personal habits of the natives, among whom his lot was cast for a number of years, so that he has been able to give us facts instead of fancies, in a plain straightforward narrative of every day scenes and occurrences, which deal with the home life of a people little understood, in the various social grades which distinguish the several classes, embracing all degrees from the poorly housed and scantily fed Coolie, to the arrogant government official. That the author has well improved his opportunities, is shown in the concluding chapters, wherein he points out the defects of a system bordering on national stagnation, and shows conclusively enough, that the greatest security of a people lies in keeping pace with the higher progress and development of the age; that indeed, no nation however populous and self reliant, can turn its back upon the achievements of modern thought in the field of art, literature and invention, and save itself from premature decay.

DISPOSAL OF WASTE AND GARBAGE.—Presented at the 19th Annual Meeting of the A. P. Health Association, Kansas City, October, 1891.

This pamphlet will be found of value to those whose duties lie in the direction of providing healthful means of disposing of the refuse which necessarily accumulates in our populous cities.

TUBERCULIN.—The value and limitation of its use in consumption. By Professor Charles Denison, A.M., M.D., Denver, Colorado.

